# Apparent Digestibility Coefficients of Black Soldier Fly (*Hermetia illucens*), Yellow Mealworm (*Tenebrio molitor*), and Blue Bottle Fly (*Calliphora vicina*) Insects for Juvenile African Catfish Hybrids (*Clarias gariepinus* × *Heterobranchus longifilis*)

**DOI:** 10.1155/2022/4717014

**Published:** 2022-10-18

**Authors:** Zsuzsanna J. Sándor, Vojislav Banjac, Strahinja Vidosavljević, Jenő Káldy, Robert Egessa, Éva Lengyel-Kónya, Rita Tömösközi-Farkas, Zsolt Zalán, Nóra Adányi, Balázs Libisch, Janka Biró

**Affiliations:** ^1^Research Centre for Aquaculture and Fisheries (HAKI), Hungarian University of Agriculture and Life Sciences, Anna liget u. 35, Szarvas, Hungary; ^2^University of Novi Sad, Institute of Food Technology, Bulevar cara Lazara br. 1, Novi Sad, Serbia; ^3^Doctoral School of Animal Husbandry Science, Hungarian University of Agriculture and Life Sciences, Gödöllő, Hungary; ^4^National Agricultural Research Organisation (NARO), Jinja, Uganda; ^5^Research Group of Food Science, Institute of Food Science and Technology, Hungarian University of Agriculture and Life Sciences, Herman Ottó u. 15, Budapest, Hungary; ^6^Institute of Genetics and Biotechnology, Hungarian University of Agriculture and Life Sciences, Szent-Györgyi Albert u. 4, Gödöllő, Hungary

## Abstract

A digestibility trial was conducted with African catfish hybrid juveniles in order to determine the apparent digestibility coefficients (ADCs) of different nutrients. The experimental diets contained defatted black soldier fly (BSL), yellow mealworm (MW), or fully fat blue bottle fly (BBF) meals, in a 70 : 30 ratio between the control diet and the tested insect meals. The indirect method for the digestibility study was performed using 0.1% yttrium oxide as an inert marker. Fish juveniles of 217.4 ± 9.5 g initial weight were distributed in 1 m^3^ tanks (75 fish/tank) of a recirculating aquaculture system (RAS), in triplicates, and fed until satiation for 18 days. The average final weight of the fish was 346 ± 35.8 g. The ADCs of the dry matter, protein, lipid, chitin, ash, phosphorus, amino acids, fatty acids, and gross energy for the test ingredients and diets were calculated. A six-month storage test was carried out to evaluate the shelf life of the experimental diets, while the peroxidation and microbiological status of the diets were also assessed. The ADC values of the test diets differed significantly (*p* < 0.001) compared to those of the control for most of the nutrients. Altogether, the BSL diet was significantly more digestible for protein, fat, ash, and phosphorus than the control diet but less digestible for essential amino acids. Significant differences were found between the ADCs of the different insect meals evaluated (*p* < 0.001) for practically all nutritional fractions analyzed. The African catfish hybrids were able to digest BSL and BBF more efficiently than MW, and the calculated ADC values agreed with those of other fish species. The lower ADCs of the tested MW meal correlated (*p* < 0.05) with the markedly higher acid detergent fiber (ADF) levels present in the MW meal and MW diet. Microbiological evaluation of the feeds revealed that mesophilic aerobic bacteria in the BSL feed were 2–3 orders of magnitude more abundant than those in the other diets and their numbers significantly increased during storage. Overall, BSL and BBF proved to be potential feed ingredients for African catfish juveniles and the shelf life of the produced diets with 30% inclusion of insect meal retained the required quality during a six-month period of storage.

## 1. Introduction

Feed manufacturers show an increasing interest in insect-derived raw materials because of their potential as fish meal substituents. The ecological footprint of insect cultivation is much lower than that of field crops, which make up the vast majority of animal feeds. The development and reproduction cycle of insects requires a short period of time. They can be grown on biowaste, and being ectotherm organisms, they have high feed utilization rates [1]. The need for valuable proteins in fish diets is increasing due to the scarcity and limited availability of marine raw material resources and the limited suitability of some terrestrial plants as ingredients of aquafeeds. Various feeding experiments with insects have been carried out using many aquaculture species in the last ten years, and the results so far are also encouraging for their industrial production [2–6]. Since the authorization of insect farming in the European Union **(**EU Regulation no. 2017/893), more than 5000 tons of insect protein have been commercialized by European insect producers [7]. According to the recommendation of the European Food Safety Authority, the following species are eligible for farming purposes: black soldier fly (*Hermetia illucens*), common housefly (*Musca domestica*), yellow mealworm *(Tenebrio molitor*), lesser mealworm (*Alphitobius diaperinus*), house cricket *(Acheta domesticus)*, banded cricket (*Gryllodes sigillatus*), and field cricket (*Gryllus assimilis*). Nevertheless, in non-EU countries, regulations are different and other insects are also considered useful for fish nutrition [8, 9].

The chemical composition and nutritional value of insect larvae are variable and depend on many factors. The most important factors are the rearing substrate and processing methodology ([2, 10]. Insect meals are high in protein and have an immunostimulatory effect due to their chitin content [9, 11–13]. Insect proteins have the advantage of high levels of the essential amino acid lysine (LYS), methionine (MET), and leucine (LEU), which are usually limiting in plants [14]. Another product made from insects is insect fat, which is favorable for fish mainly due to the presence of lauric acid, a medium-chain fatty acid considered to have antibacterial effect on fish [15]. Besides the available nutrients in the feedstuffs, knowledge of the digestibility of the various feed ingredients is also required. Together with chemical analysis, determination of digestibility concerning the nutrients and energy may allow a more thorough estimation of the nutritive value of a particular protein source in a complete feed for fish.

Although insects have the potential as good protein and fat sources, less information is known about their utilization as feed ingredients for intensively reared African catfish (*Clarias gariepinus*), an important and dominant aquaculture species in Hungary. Meals from insects such as shea caterpillar (*Cirina butyrospermi*), housefly, variegated grasshopper (*Zonocerus variegatus* L.), and black solider fly have previously been included in the diets of African catfish as alternative protein sources [16–21]. However, no data are available on the digestibility of insect meals as ingredients for this fish species.

The digestibility of feeds containing different proportions of insect meals has been examined, and the concerned studies were reviewed by Gasco et al. [22]. The most frequently investigated insects in these studies were the black soldier fly and mealworm, and finally, the optimal inclusion level for nutrition was suggested. Only a few studies estimated the digestibility of insect meals by different fish species. Among them, apparent digestibility coefficients (ADCs) of housefly maggot meal were determined for Nile tilapia (*Oreochromis niloticus*) and common carp (*Cyprinus carpio*) [23] and ADCs of several *Coleoptera*, *Orthoptera*, and *Blattodea* species in Nile tilapia fingerlings [24]. Mohamad-Zulkifli et al. [25] presented ADC values of a processed black soldier fly (BSL) meal fed to hybrid grouper (*Epinephelus fuscoguttatus ♀* × *Epinephelus lanceolatus* ♂), and ADC results for BSL are also available for Atlantic salmon (*Salmo salar*) [26].

Black soldier fly and yellow mealworm (MW) are the most frequently utilized insects in fish nutrition. BSL belongs to the Diptera, *Stratiomyidae* family and is present on all major continents, predominantly in temperate and tropic regions. It can successfully develop on vertebrate remains, kitchen waste, fruits and vegetables, raw liver, fish offal, municipal and human waste, and dairy cattle manure. MW belongs to the order Coleoptera, and it has been produced in large quantities since the 1950s, initially for fishing bait and later for pet food and for the songbird feed market. Nowadays, thousands of tons of dry mealworm are produced and sold worldwide [27–29]. The MW can grow and reproduce when fed exclusively on wheat bran or additional food supplements [30, 31]. Blue bottle fly (BBF) (*Calliphora vicina*, *Calliphoridae*, and *Diptera*), commonly referred to as “meat fly,” is used as bait or boilies ingredient for fishing or angling. However, its utilization in fish feeds is not yet authorized in EU countries (EU Regulation no. 2017/893) but it is present in several fishing products available in Europe, too.

The microbiological composition of the fish feed has a high impact on the weight gain and fish health condition [32]. A wide range of microbes occurs naturally in feeds or as contaminants of feeds. These microbes could be nonpathogenic bacteria, but often, these are molds or also harmful bacteria such as *Salmonella*, *Listeria*, and *E. coli* [33]. The insects may contain several insect-specific pathogenic microorganisms that have to be considered when the feed safety aspect is assessed. The risk of transmission from a rearing substrate to the insect can be reduced by hygienic culturing. Fortunately, the risk posed by pathogenic microorganisms is mitigated during the insect meal production process or later in the feed extrusion step. Autochthonous microbiota of the insects including bacteria, fungi, and viruses have been explored by different authors [34, 35]. However, in case of most pathogens, no active growth occurs in the intestinal tract of insects [36].

Taking into consideration the abovementioned gaps in our knowledge, we aimed to determine the apparent digestibility coefficients (ADCs) of African catfish hybrid juveniles for BSL, MW, and BBF meals. Accordingly, the present study demonstrates a short-term digestibility trial conducted in a recirculating aquaculture system (RAS) using three experimental diets containing different insect meals. In addition, the stability and shelf life of these feeds were followed up during a six-month storage period.

## 2. Materials and Methods

### 2.1. Description of the Insect Meals

The defatted black soldier fly meal was supplied by Agroloop Ltd., Netherlands; defatted yellow mealworm meal was imported from Berg and Schmidt Pte. Ltd., Singapore; while the fully fat blue bottle fly meal was produced by Csali Hungary Ltd. (Kiskunhalas, Hungary). The composition (% DM) and gross energy value (MJ·kg^−1^) of insect meals are summarized in Table 1. There was high variability in chemical composition of the ingredients. The protein content was high in BSL and MW, while in the case of BBF, the protein level was lower due to the high amount of fat in it. Crude ash, Ca, P, and chitin content were the highest in BSL compared to the others, while the acid detergent fiber (ADF) content was the highest in MW. The chitin content ranged between 5.8 and 9.6%. The ash-free ADF of BBF was the lowest (15%), while for BSL and MW, 22% and 27% were determined, respectively. The gross energy content of feed ingredients ranged from 20.7 MJ·kg^−1^ (for MW) to 25.64 MJ·kg^−1^ (for BBF). The essential amino acid content differed significantly in HIS between the meals. The highest level of LYS and MET and the lowest level of LEU have been observed in the BBF meal. Finally, the sum of essential amino acids (EAA) was the lowest in MW. The fatty acid profile of the meals differed in some cases. The lauric acid (12 : 0) content was the highest in BSL (43.12%) although the sample was defatted, while in MW and BBF, its level was very low (0.12–0.25% or 4.01 mg/100 g D.M., 0.02 mg/100 g D.M., and 0.04 mg/100 g D.M., respectively). In BBF and MW, the oleic acid (18 : 1*n* − 9) and linoleic acid (18 : 2*n* − 6) levels were about two times higher than those in the BSL sample. Consequently, total monounsaturated (MUFA) and polyunsaturated (PUFA) fatty acid levels were also high in the MW and BBF samples. Regarding the long chain n-3 polyunsaturated FAs (Lc-PUFAs), the insects do not contain them, thus depending on the rearing substrate, they can be detected only at trace levels in insects.

### 2.2. Diet Preparation for the Digestibility Trial

The nutrient content of the tested insect meals was examined in detail in order to satisfy the needs of omnivorous fish species since information on the nutrient requirements of African catfish [38] is scarcely available. The amino acid balance of these insect meals was comparable to that of fish meal [39] and was sufficient to meet the dietary requirement for catfish. However, the quantity of MET and LYS should be increased in feed formulations. Four different feeds for African catfish were produced at the Institute of Food Technology (University of Novi Sad, Novi Sad, Serbia) for digestibility trials of three different insect meals. For this purpose, a control feed (reference) was formulated, which was then mixed individually with the test ingredients at a 70 : 30 ratio (control: test, as is basis) to produce the experimental diets [40–43] and were then extruded. The control feed was prepared to be a high fish meal diet in order to be easily digestible for fish.

The dry ingredients were mixed in a double-shaft paddle mixer (model SLHSJ0.2A, Muyang, Yangzhou, China) for 120 s according to Table 2 and the experimental setup. Yttrium-oxide, as a marker for digestibility assessment, was added to each diet at a 0.1% level. Dry mixtures were processed using a twin-screw extruder (Bühler BTSK-30, Bühler, Uzwil, Switzerland) and then subsequently dried in the continuous vibro dryer, model FB 500 × 2000 (Amandus Kahl GmbH & Co., KG, Germany) at 80°C for approximately 10 minutes. The final pellets were 4.5 mm in diameter and semifloating. Proximate, fatty acid, amino acid composition, and gross energy of diets are shown in Table 2.

### 2.3. Fish Feeding and Faeces Collection

The research was carried out with African catfish hybrid (*Clarias gariepinus* × *Heterobranchus longifilis*) juveniles. The experiments were conducted according to the European Union Directive 2010/63/EU regarding the protection of animals for scientific purposes. The animal experiments and related samplings were approved by the Ethical Committee of HAKI (license no. BE/25/4302-3/2017), which was established according to the Hungarian State law 9/1999 (I. 27.), and it is operated according to the relevant Hungarian legislation concerning animal experiments, transportation of animals, and their welfare (40/2013. II. 14).

Nine hundred African catfish juveniles (average weight of 217.4 ± 9.5 g) originating from the institutional hatchery facility of HAKI were distributed in a RAS equipped with twelve 1 m^3^ fiberglass tanks (75 fish per tank). Three experimental groups and one control group were set up and randomly distributed in tank triplicates. The water flow was adjusted to an average of 4.5 L/min per tank, the dissolved oxygen level was kept above 80% saturation, ammonia-N was below 0.1 mL/L, and pH varied between 7.8 and 8.4. The water temperature was set to 23 ± 1°C. During three days of acclimatization, the fish were fed with a commercial diet and thereafter switched to experimental diets. Fish were hand fed till apparent satiation with the experimental diets 3 times per day for 18 consecutive days. On the last day of feeding, 15 individuals from the fish stock per tank were sampled in order to collect faeces from the intestine [45]. The average final weight of fish was 346 ± 35.8 g. Before harvesting, fish were anesthetized with norcaicum-/tonogen- (50 mL/100 L) based anesthesia [46]. The whole intestines were removed and the solid part of the faeces was collected as pooled samples per treatment. The fecal samples were refrigerated, freeze dried, and stored in exicator until analysis. The evaluation of growth parameters was not considered in this trial.

### 2.4. Analytical Methods

The chemical composition of test ingredients, feeds, and faeces was analyzed by standard methods of the AOAC [47]. Crude protein (CP) was determined by the Kjeldahl method [47] using digestion block (KJELDATHERM, Gerhardt, Germany) via a distillation procedure (VAPODEST 450, Gerhardt, Germany). 0.5 g dry samples were digested with 10 mL of cc H_2_SO_4_ and 10 mL of 30% H_2_O_2_. Afterwards, the generated ammonium sulphate was distilled off by using 2% H_3_BO_3_. The CP was calculated as *N* × 4.75 in the case of insects and *N* × 6.25 for diets and faeces. The crude fat was determined from the 5 g dry sample according to the AOAC 945.16 Soxhlet method using an automatic system (SOXTHERM® Unit SOX416, Gerhardt, Germany) and diethyl ether (boiling point, 40–60°C) as a solvent. The crude ash content was estimated according to the AOAC 942.05 method. Two grams of each sample were weighed, placed in a furnace, and heated at 550°C for 4 h. The amount of remaining ash was recorded. The crude fiber content was determined from defatted samples [47]. The sample amount was 1.5–2.0 grams, and the digestion procedure was carried out using 0.13 M H_2_SO_4_ and 0.313 M NaOH in a GERHARDT Fibretherm FT12 apparatus (Königswinter, Germany). The acid detergent fiber (ADF) was determined with the same equipment by using ADF solution prepared from N-cetyl-trimethyl-ammonium bromide dissolved in 0.5 M H_2_SO_4_ (100 g/5 L) and a few drops of antifoaming agent. The chitin content was determined as the difference between ash-free ADF and protein linked to ADF (ADIP) (chitin% = ADF% − ADIP%) according to Finke [48] and Marono [44]. The gross energy was determined by a Parr Instruments 6400 calorimeter bomb (Moline, Illinois, USA) calibrated with benzoic acid.

The fatty acid composition of different samples was analyzed by the capillary gas chromatographic method. Lipids were extracted from the samples with a 2 : 1 mixture of chloroform and methanol. The extracts were purified according to the method by Folch et al. [49]. Aliquots of total lipid samples were trans-esterified using a methanolic solution of HCl [50]. Fatty acid methyl esters (FAMEs) were separated on fused silica capillary columns (DB-225; Agilent) in an Agilent (HP) gas chromatograph system (AGILENT 6890N, California, USA) equipped with a flame ionization detector (FID) and a mass spectrometer detector (MSD) (Agilent, B5973N). The FAMEs were identified using authentic primary (Supelco, Bellefonte, NJ, USA) or secondary (e.g., linseed oil and cod liver oil) standards and by means of the relationship between the logarithms of relative retention times and the carbon number (Cn) of fatty acids. Fatty acid concentrations were expressed as a weight percentage of the FA sample, as assessed by the relative response factor (RRF) and molar concentration of FAME [51, 52]. Total lipids were calculated by summing the milligram per gram values of the present fatty acids in the samples.

The amino acid content of samples was analyzed using the UPLC-DAD method (Waters Acquity UPLC H-Class, Milford, USA) after acid hydrolysis and precolumn derivatization with 6-aminoquinolyl-N-hydroxysuccinimidyl carbamate (AQC) reagent. The analysis was performed with AccQ UPLC BEH C18 2.1 × 100 mm, 1.7 *μ*m column (Waters), and AccQ Tag Ultra eluents A, B, and water in the gradient mode, the flow rate being 0.7 mL/min. The chromatograms were evaluated at 260 nm, using amino acid standards. Acid hydrolysis was carried out for amino acid analysis. Twenty-five milligrams of the samples were hydrolyzed by 6 N HCl containing 1% of phenol in a Milestone Ethos One Microwave digestion system. Hydrolysates were completed to 5 mL by 1 M borate buffer (pH 8.51).

Yttrium, calcium, and phosphorus contents were analyzed by the ICP method. The digestion of samples was carried out with mixtures of acids, including nitric acid (R.G. 65%) and hydrogen peroxide (R.G. 30%). The extraction was realized by using the microwave digestion technique under high pressure and a Milestone Ethos Plus (Sorisole, Italy) microwave apparatus. The concentrations of elements were measured by Thermo Scientific 6500 ICP-OES (Massachusetts, USA) equipment.

### 2.5. Calculations and Statistical Analyses

The apparent digestibility coefficients (ADCs) of dry matter, protein, lipid, fiber, chitin, ash, phosphorus, amino acids, fatty acids, and gross energy for the test ingredients and diets were calculated as follows [40, 53].

(1) $$\begin{array}{c} {\text{ADC}}_{\text{diet}}=\left[1–\left(\left\{\frac{Y_{\text{diet}}}{Y_{\text{faeces}}}\right\}\times \left\{\frac{D_{\text{faeces}}}{D_{\text{diet}}}\right\}\right)\right]\times 100, \end{array}$$

where *Y*_diet_ is the dietary yttrium level, *Y*_faeces_ is the faeces yttrium level, *D*_diet_ is the dietary nutrient level, and *D*_faeces_ is the faeces nutrient level.

The apparent digestibility coefficients of the test ingredient (BSL, MW, and BBF) were calculated according to Bureau et al. [53] as follows:

(2) $$\begin{array}{c} {\text{ADC}}_{\text{ingredient}}=\text{AD}{\text{C}}_{\text{test diet}}+\left[\left({\text{ADC}}_{\text{test diet}}–\text{AD}{\text{C}}_{\text{contr diet}}\right)\text{ x }\left(\frac{0.7\times D_{\text{contr}}}{0.3\;D_{\text{ingredient}}}\right)\right], \end{array}$$

where *D*_contr_ is the % nutrient (or kJ g^−1^) of control diet (dry matter basis) and *D*_ingr_ is the % nutrient (or kJ g^−1^) of insect ingredient (dry matter basis).

All data are presented as means ± SD and subjected to one-way analysis of variance (ANOVA) to determine whether significant differences occurred among treatments. If a significant difference was identified, differences among means were compared with Tukey's post hoc test and two-sample *t*-test. All statistical analyses, including Pearson correlations between dietary nutrient levels in insects and ADC values, were performed by the SPSS 22 (SPSS Inc., Chicago, IL, USA) software package.

### 2.6. Shelf-Life Tests of the Experimental Feeds

The peroxidation and microbiological status of the diets were evaluated during a six-month period of storage. The experimental feeds were stored in the storage room of the fish rearing facility. Samples were collected at appropriate times and stored at −80°C until analysis. The peroxide value (POV) is defined as the reactive oxygen content expressed in terms of milliequivalents (meq) of free iodine per kilogram of fat. It is determined by titrating iodine liberated from potassium iodide with sodium thiosulphate solution. Briefly, 50 g of each feed sample was extracted with petrol ether (30–40°C) and evaporated at 40–45°C. The etheric fat solution (approx. 0.3 g fat) was vacuum evaporated and mixed with 0.5 g of KI (powdered) and 10 mL of an acetic acid and chloroform (3 : 2) solvent mixture. Titration was carried out with 0.1 M Na_2_S_2_O_3_ solution using a 1% starch indicator [47].

The mesophilic aerobic microbial cell count, the mold, *Enterobacteriaceae*, *E. coli*, *Salmonella*, and *Clostridium perfringens* cell numbers were measured to assess the microbial contamination of feeds. Samples were taken on weeks 1, 4, 8, 16, and 21 of the storage period. An aliquot of each sample (1.0 g) was weighed under sterile conditions into 9 mL peptone salt solution for the determination of mesophilic aerobic microbes and mold or into an enrichment broth in the case of *Enterobacteriaceae*, *E. coli* (EE-Mossel broth), *Salmonella* (Rappaport Vassiliadis (R.V.S.) broth), and *Clostridium* (DRCM broth). The samples were vortexed intensively for 30 s and allowed to dissolve, and from the salt solution, serial decimal dilutions were prepared and 0.1 mL of each dilution was spread onto dishes containing Dichloran Rose-Bengal Chloramphenicol (DRBC) agar for mold and Plate Count Agar (PCA) for mesophilic aerobic microbes and incubated at 25°C for 2–3 days. The enrichment broths were incubated at 35°C for 24–48 hours. For the identification of *Enterobacteriaceae*, *E. coli*, *Salmonella*, and *Clostridium* Violet Red Bile (VRB) agar, FluoroBio® VRBL agar, Harlequin™ Salmonella ABC agar, and Tryptose Sulfite Cycloserine (TSC) selective agar were used, respectively. If any of the investigated bacteria were identified from the enrichment broth, the quantitative analysis was carried out from the original sample by using the selective media. All experiments were performed in triplicates.

## 3. Results

### 3.1. Apparent Digestibility Coefficient of the Diets

The apparent digestibility coefficients of dry matter (ADCDM), crude protein (ADCPr), crude fat (ADCF) and gross energy (ADCGE), essential amino acids (ADCEAAs), fatty acids (ADCFA), phosphorus (ADCP), crude ash (ADCA), and chitin (ADCCh) of the diets were estimated based on the digestibility trial and sampling of faeces. The results of different test diets and the control diet are shown in Table 3. The ADCDM, ADCPr, and ADCGE of BSL and BBF diets did not differ significantly from the control diet, while digestibility coefficients for other nutrients, such as ADCF and ADCP, significantly differed. The ADC of each nutrient determined in the MW diet was significantly lower compared to the control diet except for phosphorus and differed from the BSL and BBF diets as well. The ADCAs were typically low and ranged between 37.01 and 61.92%, with significant differences in the case of BSL and MW compared to the control diet. The ADCEAA values ranged between 79.34 and 93.65% for the BSL diet, except ILE and THR (73.81% and 73.00%, respectively), and differed significantly from the control except ARG. The ADCEAA values obtained for the MW diet differed from the control diet in all parameters and from other testing diets as well. Significantly lower ADCEAAs were calculated for BBF in most of the cases compared to the reference diet except HIS and LYS. The ADCFAs were the highest among the tested nutrients in the range of 96.36–99.45%, except for lauric acid in the MW diet (77.81%) and control diet (82.23%). The ADCs of lauric acid in the BBF and BSL diets were significantly higher compared to the control and MW diets. Moreover, in most of the cases, ADCFAs were significantly higher for BBF compared to the control diet (except for DHA).

### 3.2. Apparent Digestibility Coefficient of the Ingredients

Following the calculated ADC values of the diets, the ADC of the test ingredients could be determined using the equations presented in Section 2.5. These data are presented in Table 4. The ADCPr for insects from the order Diptera (BSL and BBF) ranged between 76.04% and 83.93%, while 49.28% was found for MW (order Coleoptera). Similarly, the ADCFs were 93.90% and 96.41% for these species, respectively, but only 61.86% was obtained for MW. The same tendency was found for some other parameters, with a much lower value for MW. The availability of P was remarkable in the Diptera meals (ADCP between 80.61% and 94.16%), while for MW, the ADCP was about 64.16%. As for gross energy, ADCGEs for BSL and BBF were significantly (*p* < 0.001) higher than those of MW. The chitin digestibility was relatively high for BSL (96.05%) and significantly differed from other meals. In respect of the digestibility of several micronutrients of the insect meals, the ADC values are summarized in Table 4. The best digestible AAs were ARG, MET, and LYS in the range of 79.15–83.91% for BSL, 75.68–90.45% for BBF, and 33.84–60.45% for MW. In the case of MW, some data obtained had negative digestibility values after using the mathematical digestibility equations and these data were excluded. The ADC of fatty acids was generally high in all insect meals.

Pearson correlation analyses were performed between the nutrient contents of the insect meals and experimental diets and between the ADC values calculated for the insect meal ingredients. While the chitin contents of the three tested insect meals did not differ significantly (*p* > 0.05), the acid detergent fiber (ADF) levels in the MW meal and also in its experimental diet (27.69% and 10.6%, respectively) were significantly higher (*p* < 0.01) compared to the other tested insect meals and diets, respectively (see Tables 1 and 2). As shown in Table 5, Pearson correlation analyses revealed significant negative correlations between the ADC values calculated for protein, fat, phosphorus, gross energy, LYS, MET, and saturated fatty acids as insect meal ingredients and between the ADF levels of the experimental diets and in some cases also between the ADF levels of insect meals (for ADCLYS, ADCMET, and ADC18 : 0).

### 3.3. Shelf-Life Tests of the Diets

**Figure 1:**
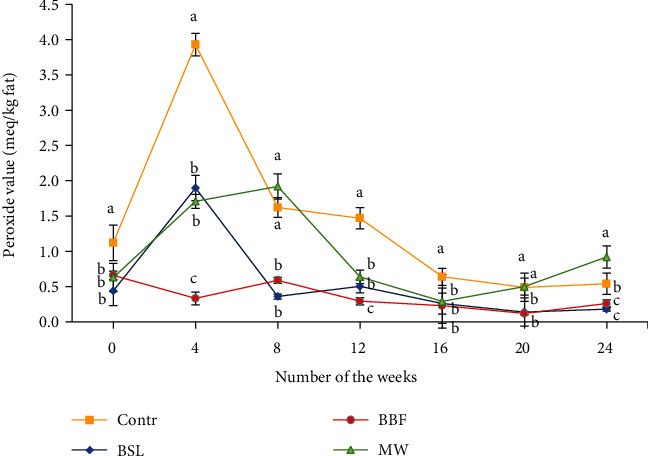
The peroxide value of the diets (meq/kg fat) during a 6-month storage period. The statistical IDs marked with lowercase letters translate into a difference between treatments at the same time at the significance level of *p* < 0.05.

The microbiological and peroxidation status of the insect meal supplemented feeds used in the fish trial was monitored during a six-month storage period. Significant differences were found in peroxide values (POVs) between the control diet and the experimental diets after four weeks of storage, where the highest value was detected in the control diet (3.98 ± 0.16 meq/kg fat). Moreover, the POV of the BBF diet was significantly lower than those of the BSL and MW diets (Figure 1). The highest POV was measured in the control diet after 12 and 16 weeks of storage as well. The BBF-containing feed showed the lowest oxidation during storage, having only 0.66 ± 0.06 meq/kg fat as the highest value.

**Figure 2:**
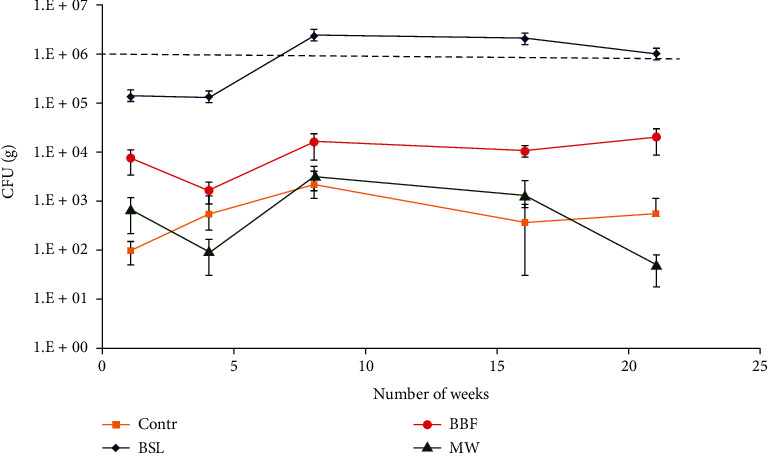
Mesophilic aerobic microbial cell count changes over time.

During the microbiological evaluation of the feeds, the total number of mesophilic aerobic bacteria was examined by repeated sampling for 21 weeks (Figure 2). The average colony forming unit (CFU) of the feeds was initially 10^2^–10^3^, except for the BSL feed, which was outstanding in terms of the number of mesophilic aerobic bacteria, containing 2–3 orders of magnitude of more microorganisms than the other **s**amples. Nevertheless, there was a significant difference between the samples (*p* < 0.05) except for MW and CONTR. During the 21-week storage period, the total mesophilic aerobic bacterial cell counts did not change much from a microbiological point of view, as only one order of magnitude increase or decrease was observed, but for BBF and BSL, the cell numbers at the end of the storage were statistically significantly higher (*p* < 0.05) compared to the first week. The cell numbers in the MW and CONTR samples did not change significantly during the storage, although a slight decrease was observed in the MW sample (Table 6).

In a more detailed examination, the number of molds and the *Enterobacteriaceae*, *Salmonella* spp., *E. coli*, and *Clostridium perfringens* appearance was investigated from samples taken at the beginning and at the end of the storage experiment. Based on these results, it can be concluded that the number of molds did not change during the study and they were in the undetectable range or just reached the 10^2^ mold/g level, while members of *Enterobacteriaceae* appeared in BBF feed in small numbers. *Salmonella* spp. (<25 CFU/g), *E. coli*, and *Clostridium perfringens* were not found (<10^2^ CFU/g) in the samples during the storage period.

## 4. Discussion

### 4.1. Digestibility of the Insect Meals

The digestibility of the feed ingredients and the availability of nutrients are the most important factors in fish nutrition. In the current study, the ADCs of three possible insect protein sources were assessed in a digestibility trial for African catfish juveniles.

BSL is one of the most frequently studied insects in fish nutrition. In the current study, the ADCs of dry matter, crude protein, and gross energy in the BSL diet were not significantly different from the reference control feed, while others such as ADCF and ADCP were significantly higher compared to the control (Table 3). The ADCPr of the test BSL diet (83.47%) in our study was lower than those reported for BSL meal in European sea bass (*Dicentrarchus labrax*) (91–93%) ([54], rainbow trout (*Oncorhynchus mykiss*) (87–91%) [55], and Atlantic salmon (90%) [26], but it was similar to turbot (*Psetta maximus*) (81.1%) [56] and close to the 86% found for Siberian sturgeon (*Acipenser baerii*) [5]. African catfish also showed similar digestibility regarding crude protein (81.2%), lipid (89.8%), and dry matter (74%) [57] when cricket meal (*Gryllus bimaculatus*) was fed. In terms of ADC of the ingredients, the protein digestibility of the BSL is in line with data presented for hybrid grouper (81–88%) [25], higher than for turbot (63.1%) [56] and lower than for African catfish fingerlings (85–91%, depending on feeding regime) [43], and Atlantic salmon (89%) [26].

The lipid ADC was higher than the protein digestibility value. Lipids are a preferable energy source to carbohydrates and are almost completely digestible by fish. The high ADCF indicates a strong ability of African catfish to utilize the lipid components of insects. Comparable high ADCFs of the diets and ingredients have been reported for Atlantic salmon, rainbow trout, turbot, and hybrid grouper [25, 26, 55, 56]. The ADC of gross energy (ADCGE) in the diet with BSL inclusion agrees with the results of the abovementioned publications except for rainbow trout where only 60–65% was reported [55]. Considering BSL as ingredient, the ADCGE in African catfish was generally higher than that in turbot (54.5%) [56] or maggot in carp and tilapia (74.9% and 58.1%) [23]. This suggests that BSL is a promising ingredient in relation to energy utilization in African catfish juveniles.

The ADCs of amino acids in the diet were comparable to those reported for Atlantic salmon [58], rainbow trout [59], and European seabass [54] with different inclusion levels of BSL. The ADCAA decreased with BSL inclusion in the diet of Atlantic salmon in the study by Belghit et al. [60], but this reduction did not affect the growth performance of the fish or feed conversion ratio, and finally, it was concluded that the BSL was still highly digestible for Atlantic salmon. The ADC of arginine was the highest among the EAAs in our study, demonstrating its high bioavailability in BSL meal. This observation is in line with results presented for Atlantic salmon [61]. Higher ADCAA was reported for the BSL ingredient in rainbow trout [59] or channel catfish (*Ictalurus punctatus*) compared to our results obtained for African catfish. However, investigated insects are still more digestible than several plants such as maize in the case of striped catfish (*Pangasianodon hypophthalmus*) [62] and sunflower meal in the case of African catfish [43]. Regarding the ADCFAs, Belghit et al. [15, 61] demonstrated highly available digestible FA in the BSL-based diets for Atlantic salmon. In our study, the ADCFAs in the test diet in most of the cases were significantly higher compared to the reference control diet, indicating that the BSL contains well digestible FAs. The level of Lc-PUFA was below the limit of detection in BSL meal, making the calculation of ADC meaningless.

All ADC values determined for the MW diet were the lowest among the tested diets in this study and differed significantly from the control diet in respect of all investigated macro- and micronutrients (Table 3). Based on our findings, it seems that the tested MW is less suitable for African catfish juveniles; however, a study by Ng et al. [63] demonstrated that MW used as insect meal is a potential protein source for this fish species. It was found to be highly palatable and could replace up to 40% of the fish meal component in diets for African catfish without any significant reduction in growth performance and feed efficiency ratio. In the case of meagre (*Argyrosomus regius*), a limited capacity to utilize MW was found, with a 10% dietary inclusion already resulting in significant impairment of fish digestive capacity and growth performance [64]. There are some studies where ADC data were investigated. For example, Chemello et al. [65] reported coefficients of total tract apparent digestibility (CTTAD) of MW supplemented diets for rainbow trout, where ADCPr was between 97 and 98%, while for gilthead sea bream (*Sparus aurata*), 79–87% was determined by Piccolo et al. [66]. These values are higher compared to our findings probably due to the different methodologies applied. Also, a higher (93%) ADCPr value was obtained by Rema et al. [67] for rainbow trout compared to ours. In terms of MW as ingredient, the ADC values for protein and crude fat (Table 4) were much lower compared to tilapia (85.4% and 90.6%) reported by Fontes et al. [24].

The ADC data calculated for the BBF diet were not significantly different from the control diet in respect of dry matter, crude protein, crude ash, and gross energy. At the same time, a significant increase was found for ADCF and ADCP. Generally, ADCs of BBF meal were significantly lower than those of BSL, but higher than those of MW. Compared with other feed ingredients, the ADCPr data indicates that BBF meal is better digested than plant feedstuffs. Such ADC data were reported for several catfish species like striped surubim (*Pseudoplatystoma reticulatum*) [68] or striped catfish [62] except for soybean meal which has the highest value among the plant products. Among the tested insects, BBF has the highest ADCAA except for arginine and leucine. Although BSL contained an appropriate level of lauric acid, a similar level was not detected in BBF even though both belong to the order Diptera. In contrast, the investigated BBF meal contained the highest Lc-PUFA level compared to BSL and MW. To the best of our knowledge, this is the first report on the ADC of BBF meal in fish and our ADC data are well comparable to other examined insects in fish nutrition. Considering our study, BBF could be a relevant protein and oil source for diet formulation.

The digestibility of nutrients may depend on other components also present in insects. One such compound is chitin, a nondigestible fiber, that is, a polymer of N-acetyl-glucosamine with *β*-(1/4) linkages. Chitin is known to interfere with protein use [69]. Based on the analytical results, the amount of chitin in the tested insect meals in our study agrees with other insects' data. Piccolo et al. [66] found 4.6% chitin (as fed) for MW, but 12% (dry matter) is reported by Fontes et al. [24] and 13.7% (dry matter) by Finke [48]. In this study, the estimated range of the chitin level in the several insect meals was between 1.16% and 13.72%.

The role of chitin in feed digestion may be influenced by several factors, considering that chitin has an immunostimulatory effect to the intestine [13, 70] and chitin was also shown to stimulate bile acid excretion resulting in an increased fecal loss of bile acids [71]. Compared to BSL, MW presents a more complex chitin-protein matrix [44] and lower trypsin susceptibility [72]. The chitin-bound nitrogen in mealworms is about 5–6% of total nitrogen [73]. Even though this is only a relatively small amount, it would still be translated into a slight decrease of available dietary protein. Nevertheless, the chitin levels of the three insect meals applied in the current study were not significantly different (*p* > 0.05) from each other, suggesting that the chitin level was not the primary factor influencing the markedly lower ADCs obtained for the MW meal.

The digestibility of chitin was determined in the current study, together with other nutrients. The ADCs for chitin (Table 4) show that African catfish can digest chitin from the investigated insect meals in different ratios. Moreover, these results are comparable with ADCCh values obtained in tilapia [24]. Although the chitinase activity was not measured in our study, many fish species, including carnivorous ones, are assumed to be unable to digest chitin [9]. Chitinase activity was detected in some fish species, but chitinolytic action seems to be limited or completely absent for most fish [56, 74–76]. Chitinolytic activity was measured in the intestine and stomach of African catfish juveniles fed on mopane worm (*Imbrasia belina*) meal; however, the results showed no discernible trend with increased mopane worm inclusion [77].

Whole insects contain variable but significant amounts of fiber as measured by ADF, although the components that make up the ADF fraction have not yet been fully characterized [44]. Finke [48] reported that the fiber content of insects measured as ADF consists chitin with significant amounts of associated cuticular proteins. The acid detergent fiber (ADF) level of MW diet in our study was high (10.1% as fed) compared to the control feed (2.13% as fed, *p* < 0.01). The ADF content of MW meal was much higher (27.7% d. m.) than the 7–11% and 7.2% reported by Marono et al. [44] and Piccolo et al. [66], respectively.

Many studies have shown that as ADF increases, digestibility and nutrient availability decreases [78]. Crude protein digestibility was negatively correlated (*p* < 0.05) to the ADF content in an *in vitro* digestibility study of *T. molito*r and *H. illucens* insect meals [44]. Pearson correlation analyses showed that the ADF level of insect meals was associated with a lower *in vitro* digestibility of organic matter (*R* = −0.59; *p* < 0.05) and lower *in vitro* digestibility of crude protein (*R* = −0.68; *p* < 0.01) in experiments using crude enzyme extracts from digestive tracts of meat-type ducks [79]. Similar observations were made in the current study where calculated ADC values negatively correlated with the ADF level of the insect meals and experimental diets (Table 5), indicating that their ADF fraction could inhibit the digestion process thereby contributing to the limited MW digestibility. Likewise, high ADF levels in mopane worm meal have previously been proposed to reduce insect meal digestibility in a feeding experiment of African catfish (*Clarias gariepinus*) juveniles [77].

Another factor that may impair insect protein digestibility is the release of insects' proteases and phenoloxidases during the grinding of whole insects [72]. Phenoloxidases are responsible for the formation of crosslinked structures between o-quinone and AA, which may negatively affect protein digestibility and digestive enzyme activities. This suggests that besides the insects' fiber content, other insect components, especially at the enzymatic level, may also influence the overall insect digestibility [64].

### 4.2. Shelf-Life Tests

The intestinal tract of insects harbors high numbers of microorganisms, which play an important role in the insects' life activity, mainly in the digestion of feed [80, 81]. The average total microbial cell counts of insects are generally high, including total mesophilic aerobes (3.6–9.4 logCFU/g), *Enterobacteriaceae* (4.2–7.8 logCFU/g), bacterial endospores or spore-forming bacteria (0.5–5.8 logCFU/g), lactic acid bacteria (LAB) (5.2–9.1 logCFU/g), psychrotrophic aerobes (4.5–7.2 logCFU/g), and yeasts and molds (3.4–7.2 logCFU/g) [82]. However, between distinct insect types, there can be a great difference in the size and composition of the microbial community. Moreover, the microbiota of insects is greatly influenced by the feed supply, rearing process, and practices [80, 81]. De Smet et al. [83] reported that microorganisms occurring in the feed can also be present in the microbial community of the insects and their diversity was found to be linked to nutritional complexity. Besides, there is a unique “core” component of the gut microbiota for every species but it may also vary with location and with feed type [83–85]. After the postharvest treatments, the processed insects generally show a lower microbial count than the fresh ones [82]. In a two-year study where fish feeds of different origin were investigated, Petreska et al. [32] found high numbers of total bacteria, followed by yeast and molds and *E. coli* to a lesser extent, which is similar to our results. The observed differences between the microbial characteristics of the investigated samples might be explained by the distinct insect types, geographic locations, and different rearing and postharvest processes. During storage, a fraction of the initially present microbial species will become dominant [80]. Vandeweyer et al. [86] have found that after a postharvest heat treatment, the microbial numbers of crickets remained constant over a 6-month storage experiment even at ambient temperature, which is similar to our results.

The insect microbiota is complex and contains a great variety of different microorganisms due to the abovementioned effects. These and the postharvest processes of feed production could play an important role in the distinct results obtained for feeds [87]. However, it should also be mentioned that in the MW feed, the total microbial colony forming units were by one order of magnitude lower compared to the control feed.

The oxidation of fat in the experimental diets was low during the storage period compared to rancid oils [88], despite that the fat content of the diets was different from each other. None of these results indicated excessive fat oxidation and deterioration of the diets after a six-month period of storage.

## 5. Conclusions

The apparent digestibility coefficients determined for the BSL meal in African catfish hybrid juveniles agreed with those reported earlier for other fish species. Based on our digestibility experiments, the BBF meal also seems to be well digestible for African catfish, similarly to BSL. However, significantly lower digestibility coefficients were obtained for the MW meal in the current study. An interaction effect between the digestibility of various insect meal ingredients and between the ADF levels was indicated by correlation analyses. Overall, our data suggest that replacing fish meal with up to 30% BSL or BBF meal in the African catfish diet would not cause difficulties in the digestibility and utilization of nutrients. On the other hand, the tested MW meal proved less suitable for African catfish, partly due to its high ADF content and possibly also due to certain enzymes that may occur among its ingredients. From the feed safety aspects, the BSL containing diet showed a potential for increasing numbers of mesophilic aerobic bacteria during storage that would require further microbiological characterization of this type of diet, regarding its origin, production, and postharvest processes.

## Figures and Tables

**Table 1:** Composition of the tested insect meals (dry weight, %).

Ingredients	BSL	MW	BBF
Dry matter	93.19 ± 0.61	91.96 ± 0.30	95.15 ± 0.16
Crude protein^∗^	52.46 ± 0.42	56.53 ± 0.14	42.54 ± 0.25
Crude fat	9.29 ± 0.40	6.20 ± 0.17	29.41 ± 0.04
Crude ash	7.80 ± 0.25	7.01 ± 0.05	4.75 ± 0.12
Crude fiber	4.81 ± 1.01	3.43 ± 0.13	5.30 ± 0.25
Phosphorus	1.01	0.54	0.78
Calcium	3.64	0.65	0.26
Gross energy (MJ·kg^−1^)	22.76 ± 0.12	20.72 ± 0.03	25.64 ± 0.07
Acid detergent fiber (ADF)	22.10 ± 0.92	27.69 ± 0.12	15.11 ± 0.33
Chitin	9.62 ± 2.01	5.81 ± 2.08	8.05 ± 1.50

*Amino acids (%)*			
Arginine (ARG)	3.30	3.54	3.05
Histidine (HIS)	1.46	0.91	1.99
Isoleucine (ILE)	3.03	2.18	2.35
Leucine (LEU)	5.02	5.22	3.84
Lysine (LYS)	3.89	3.81	4.43
Methionine (MET)	1.26	1.19	1.46
Threonine (THR)	2.78	2.37	2.66
Phenylalanine (PHE)	3.17	2.38	4.13
Tryptophan (TRP)	0.58	0.42	0.35
Valine (VAL)	3.96	3.35	3.23
ΣEAA	*32.09*	*27.62*	*31.23*
ΣAA	*55.76*	*52.61*	*54.62*

*Fatty acids (FA) (%)*			
12 : 0	43.12 ± 1.53	0.25 ± 0.01	0.12 ± 0.04
14 : 0	8.05 ± 0.45	1.09 ± 0.02	1.73 ± 0.00
16 : 0	11.65 ± 0.20	18.14 ± 0.05	18.12 ± 0.13
16 : 1*n* − 9	0.29 ± 0.00	0.28 ± 0.01	5.12 ± 0.14
16 : 1*n* − 7	3.22 ± 0.09	1.96 ± 0.03	10.79 ± 0.00
18 : 0	1.69 ± 0.16	7.52 ± 0.06	2.64 ± 0.16
18 : 1*n* − 9	15.52 ± 1.03	33.83 ± 0.10	30.36 ± 0.14
18 : 2*n* − 6	12.38 ± 0.52	27.44 ± 0.28	24.60 ± 0.34
18 : 3*n* − 3	0.57 ± 0.01	1.17 ± 0.17	0.65 ± 0.04
20 : 4*n* − 6	0.07 ± 0.00	0.27 ± 0.02	1.68 ± 0.00
20 : 5*n* − 3 (EPA)	0.00 ± 0.00	0.00 ± 0.00	0.20 ± 0.01
22 : 6*n* − 3 (DHA)	0.00 ± 0.00	0.69 ± 0.01	0.00 ± 0.00
SFA	65.68 ± 1.63	28.85 ± 0.09	22.91 ± 0.06
MUFA	19.67 ± 1.14	39.65 ± 0.12	47.54 ± 0.26
PUFA	14.58 ± 0.48	29.86 ± 0.10	28.73 ± 0.20
Total lipid (mg·g^−1^)	59.27 ± 2.81	15.77 ± 0.89	192.75 ± 1.78

^∗^Protein was calculated by applying a nitrogen to protein conversion factor of Kp = 4.76 [37].

**Table 2:** Formulation (g·kg^−1^), proximate composition (%, wet weight), gross energy (MJ·kg^−1^, wet weight) amino acid and fatty acid profile (w %) of the control, and experimental diets used in the digestibility experiment.

Ingredients	CONTR diet	BSL diet	MW diet	BBF diet
Fish meal^1^	399	280	280	280
Winter wheat^2^	330	230	230	230
Soybean protein concentrate^3^	130	91	91	91
Corn gluten^4^	110	77	77	77
Vitamin/mineral premix^5^	30	21	21	21
Insect meal^6^	0	300	300	300
Yttrium-oxide^7^	1	1	1	1

*Proximate composition %* (mean ± SD)				
Dry matter	95.69 ± 0.02	95.30 ± 0.06	96.87 ± 0.06	96.05 ± 0.01
Crude protein^∗^	47.13 ± 0.14	50.10 ± 1.00	53.14 ± 0.75	48.96 ± 0.60
Crude fat	5.80 ± 0.12	8.50 ± 0.05	5.90 ± 0.45	12.10 ± 0.79
Crude fiber	1.43 ± 0.07	4.05 ± 0.03	1.70 ± 0.01	3.55 ± 0.17
Crude ash	9.01 ± 0.04	8.33 ± 0.04	8.34 ± 0.04	7.66 ± 0.01
Phosphorus	1.03 ± 0.01	0.89 ± 0.01	0.85 ± 0.02	0.94 ± 0.03
Calcium	1.79 ± 0.01	1.98 ± 0.00	1.35 ± 0.00	1.40 ± 0.07
Gross energy (MJ·kg^−1^)	18.63 ± 0.06	19.92 ± 0.04	19.23 ± 0.02	20.66 ± 0.04
Acid detergent fiber (ADF)	2.13 ± 0.33	6.11 ± 0.76	10.06 ± 0.47	6.34 ± 0.12
Chitin^8^	0.13 ± 0.05	4.91 ± 0.54	2.94 ± 0.56	3.06 ± 0.37

*Essential amino acid (EAA) (%)*				
Arginine (ARG)	2.59	2.80	3.01	2.79
Histidine (HIS)	0.97	1.12	1.01	1.28
Isoleucine (ILE)	1.82	2.01	1.83	1.96
Leucine (LEU)	4.01	3.98	4.22	3.91
Lysine (LYS)	4.35	4.61	4.45	5.29
Methionine (MET)	0.89	0.94	0.93	1.01
Threonine (THR)	1.88	2.05	2.02	2.09
Phenylalanine (PHE)	2.21	2.36	2.42	2.82
Tryptophan (TRP)	3.07	3.07	3.64	3.38
Valine (VAL)	2.19	2.70	2.46	2.46
ΣEAA	*23.99*	*25.63*	*25.99*	*27.00*
ΣAA	*42.88*	*45.49*	*46.16*	*47.22*

*Fatty acids (FA) w %* (mean ± SD)				
12 : 0	0.08 ± 0.00	20.79 ± 0.25	0.09 ± 0.00	0.29 ± 0.02
14 : 0	4.33 ± 0.02	6.22 ± 0.02	3.87 ± 0.03	2.44 ± 0.00
16 : 0	18.20 ± 0.66	14.91 ± 0.07	18.15 ± 0.03	18.26 ± 0.04
16 : 1*n* − 9	0.29 ± 0.01	0.31 ± 0.01	0.32 ± 0.01	3.47 ± 0.00
16 : 1*n* − 7	4.51 ± 0.00	3.81 ± 0.02	4.12 ± 0.03	8.76 ± 0.04
18 : 0	2.83 ± 0.01	2.25 ± 0.03	3.61 ± 0.01	2.79 ± 0.03
18 : 1*n* − 9	15.54 ± 0.04	15.45 ± 0.10	18.57 ± 0.04	26.35 ± 0.12
18 : 2*n* − 6	12.04 ± 0.07	12.34 ± 0.12	14.65 ± 0.10	20.59 ± 0.12
18 : 3*n* − 3	1.67 ± 0.06	1.12 ± 0.01	1.56 ± 0.02	0.96 ± 0.02
20 : 4*n* − 6	0.48 ± 0.01	0.26 ± 0.00	0.46 ± 0.00	1.29 ± 0.00
20 : 5*n* − 3 (EPA)	7.08 ± 0.01	3.75 ± 0.05	5.89 ± 0.03	2.07 ± 0.05
22 : 6*n* − 3 (DHA)	12.43 ± 0.03	6.27 ± 0.12	10.24 ± 0.12	3.33 ± 0.04
SFA	26.86 ± 0.03	45.47 ± 0.16	27.24 ± 0.07	24.47 ± 0.04
MUFA	31.34 ± 0.02	25.34 ± 0.16	32.69 ± 0.09	42.63 ± 0.19
PUFA	38.08 ± 0.02	26.51 ± 0.11	36.71 ± 0.01	29.88 ± 0.03
EPA + DHA	19.51 ± 0.02	10.02 ± 0.07	16.14 ± 0.09	5.40 ± 0.10
Total lipid (mg·g^−1^)	37.15 ± 0.80	47.79 ± 0.33	30.57 ± 0.07	90.10 ± 1.03

^1^999 LT Fish meal, TripleNine Fish Protein A/S, Esbjerg, Denmark. ^2^Supplied from a local feed mill, Novi Sad, Serbia. ^3^Tradkon SPC500-P, Sojaprotein, Bečej, Serbia. ^4^Starch Industry, Jabuka DOO, Pančevo, Serbia. ^5^Ravago Chemicals (Feketić, Serbia). ^6^BSL: black soldier fly larvae supplied by Agroloop Ltd.; MW: yellow mealworm from Berg and Schmidt Pte. Ltd., Singapore; BBF: blue bottle fly produced by Csali Hungary Ltd. ^7^Alfa Aesar, Thermo Fisher (Kandel) GmbH, Karlsruhe, Germany. ^8^Chitin% = ash free ADF% − ADF protein% following the method presented by Marono [44]. ^∗^Protein was calculated by applying a nitrogen to protein conversion factor of Kp = 6.25.

**Table 3:** Apparent digestibility coefficients (ADC) of the diets prepared by using different insects as test ingredients.

ADC (%)	Control diet	BSL diet	MW diet	BBF diet	*p* value
Dry matter	79.18 ± 0.37^ab^	79.60 ± 0.25^a^	69.17 ± 0.58^c^	78.76 ± 1.20^b^	<0.001
Crude protein	83.28 ± 0.55^ab^	83.47 ± 0.06^a^	72.07 ± 0.77^c^	81.32 ± 0.76^b^	<0.001
Crude fat	89.70 ± 0.24^c^	91.86 ± 0.10^b^	81.24 ± 0.35^d^	94.23 ± 0.32^a^	<0.001
Crude ash	55.27 ± 1.65^b^	61.92 ± 0.10^a^	37.01 ± 0.15^c^	55.16 ± 2.25^b^	<0.001
Phosphorus	65.07 ± 1.32^c^	73.39 ± 1.57^a^	64.91 ± 1.13^c^	68.74 ± 1.42^b^	<0.001
Gross energy	81.82 ± 0.53^a^	82.61 ± 0.09^a^	72.21 ± 0.63^b^	81.59 ± 1.01^a^	<0.001

Essential amino acids (EAA)					
Arginine	89.27 ± 0.23^a^	87.43 ± 0.21^a^	69.35 ± 0.80^c^	84.85 ± 0.33^b^	<0.001
Histidine	84.96 ± 0.32^a^	79.34 ± 0.35^b^	61.87 ± 0.99^c^	83.24 ± 0.37^a^	<0.001
Isoleucine	82.83 ± 0.36^a^	73.81 ± 0.44^c^	49.51 ± 1.31^d^	77.24 ± 0.50^b^	<0.001
Leucine	87.89 ± 0.26^a^	79.57 ± 0.34^b^	59.67 ± 1.05^c^	81.13 ± 0.41^b^	<0.001
Lysine	90.73 ± 0.20^a^	87.62 ± 0.21^b^	80.18 ± 0.51^c^	90.65 ± 0.21^a^	<0.001
Methionine	90.93 ± 0.19^a^	87.86 ± 0.20^b^	80.14 ± 0.52^c^	89.01 ± 0.24^b^	<0.001
Threonine	81.06 ± 0.28^a^	73.00 ± 0.45^b^	45.20 ± 1.42^c^	74.95 ± 0.55^b^	<0.001
Phenylalanine	91.55 ± 0.18^a^	86.71 ± 0.22^c^	80.22 ± 0.51^d^	89.09 ± 0.24^b^	<0.001
Tryptophan	95.17 ± 0.10^a^	93.65 ± 0.11^b^	90.57 ± 0.24^c^	90.37 ± 0.12^d^	<0.001
Valine	86.15 ± 0.29^a^	78.38 ± 0.36^b^	54.20 ± 0.36^c^	82.82 ± 2.37^b^	<0.001

Fatty acids (FA)					
12 : 0	82.23 ± 2.27^b^	99.97 ± 0.00^a^	77.81 ± 2.32^b^	98.02 ± 0.19^a^	<0.001
14 : 0	98.63 ± 0.05^b^	99.22 ± 0.09^a^	98.06 ± 0.14^c^	99.03 ± 0.01^a^	<0.001
16 : 0	97.66 ± 0.23^c^	98.29 ± 0.04^b^	97.69 ± 0.03^c^	98.96 ± 0.03^a^	<0.001
16 : 1*n* − 7	98.12 ± 0.05^b^	98.08 ± 0.07^b^	98.60 ± 0.02^b^	98.78 ± 0.04^a^	<0.001
18 : 0	97.07 ± 0.56^b^	97.64 ± 0.09^bc^	96.74 ± 0.05^a^	98.65 ± 0.05^ac^	0.009
18 : 1*n* − 9	97.52 ± 0.75^b^	98.48 ± 0.13^ab^	98.00 ± 0.01^ab^	99.32 ± 0.05^a^	0.036
18 : 2*n* − 6	96.36 ± 0.68^b^	98.43 ± 0.13^a^	97.85 ± 0.06^a^	99.18 ± 0.04^a^	0.006
18 : 3*n* − 3 (LNA)	97.30 ± 0.76	98.28 ± 0.19	98.00 ± 0.20	98.38 ± 0.13	0.169
22 : 6*n* − 3 (DHA)	99.36 ± 0.05^a^	99.20 ± 0.01^a^	99.30 ± 0.01^a^	98.90 ± 0.07^b^	0.003
EPA + DHA	99.45 ± 0.05^a^	99.37 ± 0.00^a^	99.36 ± 0.03^a^	99.20 ± 0.05^b^	0.091

The statistical IDs marked with different letters within the same row translate into a deviation on a significance level of *p* < 0.05.

**Table 4:** Apparent digestibility coefficients of nutrients, gross energy, and chitin of the tested insect meals.

ADC (%)	BSL	MW	BBF	*p* value
Dry matter	80.59 ± 0.83^a^	44.86 ± 1.93^c^	77.76 ± 4.02^b^	<0.001
Crude protein	83.93 ± 0.19^a^	49.28 ± 2.39^c^	76.04 ± 2.82^b^	<0.001
Crude fat	93.90 ± 0.19^b^	61.86 ± 1.51^c^	96.41 ± 0.48^a^	<0.001
Crude ash	80.66 ± 0.40^a^	N/A	54.66 ± 9.67^b^	<0.001
Phosphorus	94.16 ± 5.49^a^	64.16 ± 6.44^c^	80.61 ± 6.02^b^	<0.001
Gross energy	84.10 ± 0.26^a^	52.05 ± 1.96^b^	81.20 ± 2.71^a^	<0.001
Chitin	96.05 ± 0.37^a^	72.84 ± 7.57^b^	68.18 ± 5.69^b^	<0.001

Essential amino acids (EAA)				
Arginine	83.91 ± 0.61^a^	33.84 ± 2.21^c^	75.68 ± 1.02^b^	<0.001
Histidine	69.80 ± 0.93^b^	N/A	81.10 ± 0.83^a^	<0.001
Isoleucine	60.61 ± 1.08^b^	N/A	66.66 ± 1.45^a^	<0.001
Leucine	63.39 ± 1.01	N/A	63.92 ± 1.47	<0.511
Lysine	79.15 ± 0.77^b^	50.85 ± 0.56^c^	90.45 ± 0.70^a^	<0.001
Methionine	82.56 ± 0.56^b^	60.45 ± 1.46^c^	86.16 ± 0.60^a^	<0.001
Threonine	59.85 ± 1.20^b^	N/A	64.55 ± 1.02^a^	<0.001
Phenylalanine	78.48 ± 0.60^b^	54.58 ± 1.68^c^	85.87 ± 0.55^a^	<0.001
Tryptophan	74.03 ± 1.49^b^	N/A	77.53 ± 2.76^a^	<0.001
Valine	67.89 ± 0.85^b^	N/A	69.30 ± 1.18^a^	<0.031

Fatty acids (FA)				
10 : 0	99.26 ± 0.22^a^	62.86 ± 2.77^b^	N/A	<0.001
12 : 0	100.01 ± 0.01^b^	70.61 ± 6.22^c^	102.51 ± 1.82^a^	<0.001
14 : 0	99.64 ± 0.11^a^	84.66 ± 1.28^b^	99.59 ± 0.06^a^	<0.001
16 : 0	99.61 ± 0.17^a^	97.85 ± 0.18^b^	99.50 ± 0.03^a^	<0.001
18 : 0	98.91 ± 0.09^b^	96.07 ± 0.17^c^	99.35 ± 0.10^a^	<0.001
18 : 1*n* − 9	99.75 ± 0.28	99.16 ± 0.07	99.70 ± 0.06	<0.068
18 : 1*n* − 7	99.78 ± 3.64^b^	102.47 ± 0.32^a^	99.43 ± 0.07^b^	<0.001
18 : 2*n* − 6	101.09 ± 0.32^a^	101.29 ± 0.37^a^	99.75 ± 0.05^b^	<0.001
18 : 3*n* − 3	102.11 ± 1.00^a^	103.18 ± 1.13^a^	99.54 ± 0.33^b^	<0.001
22 : 6*n* − 3	N/A	90.16 ± 0.22	N/A	—
EPA + DHA	N/A	84.94 ± 3.07^b^	89.08 ± 1.41^a^	<0.001

N/A: not applicable. N/A values assigned when nutrient levels in the ingredient were traced, resulting in a negative digestibility value using the mathematical digestibility equation. The statistical IDs marked with different letters translate into a significant difference at the level of *p* < 0.05.

**Table 5:** Pearson correlation coefficients between ADCs of insect meal ingredients and ADF levels in the tested insect meals and diets.

	ADCPr	ADCF	ADCP	ADCGE	ADCLYS	ADCMET	ADC12:0	ADC16:0	ADC18:0
ADF in IM^a^	−0.575	−0.806	−0.274	−0.686	*−0.915* ^b^	*−0.845* ^b^	−0.804	−0.739	*−0.847* ^b^
ADF in diet	*−0.964* ^c^	*−0.979* ^c^	*−0.84* ^b^	*−0.983* ^c^	*−0.931* ^c^	*−0.971* ^c^	*−0.976* ^c^	*−0.969* ^c^	*−0.945* ^c^

^a^IM: insect meal. ^b^Correlations significant at the *p* < 0.05 level. ^c^Correlations significant at the *p* < 0.01 level.

**Table 6:** Microbiological evaluation of the diets during the storage period (CFU·g^−1^ feed).

Feeds	Mesophilic aerobic microbes		*Enterobacteriaceae*	
Tolerance threshold	10^6^ CFU·g^−1^		10^3^ CFU·g^−1^	
Week	1^st^	21^st^	1^st^	21^st^
MW	700 ± 480^cC^	50 ± 33^cC^	0	0
BBF	7600 ± 4400^bB^	21000 ± 11000^bA^	<100	<100
BSL	140000 ± 410000^aB^	1100000 ± 300000^aA^	0	0
CONTR	100 ± 52^cC^	550 ± 220^cC^	0	0

Tolerance threshold according to the 65/2012. (VII. 4.) Hungarian Ministry of Rural Development regulation. Different lowercase letters within a column indicate a significant difference between the samples according to the two-sample *t*-test (*p* < 0.05). Different uppercase letters within a row indicate a significant difference between the storage time within the same sample according to the two-sample *t*-test (*p* < 0.05).

## Data Availability

All data generated or analyzed during this study are included in this published article.
